# ExPheWas: a platform for *cis*-Mendelian randomization and gene-based association scans

**DOI:** 10.1093/nar/gkac289

**Published:** 2022-04-26

**Authors:** Marc-André Legault, Louis-Philippe Lemieux Perreault, Jean-Claude Tardif, Marie-Pierre Dubé

**Affiliations:** Montreal Heart Institute, Montreal, QC H1T 1C8, Canada; Université de Montréal Beaulieu-Saucier Pharmacogenomics Centre, Montreal, QC H1T 1C8, Canada; Department of Biochemistry and Molecular Medicine, Faculty of Medicine, Université de Montréal, Montreal, QC H3T 1J4, Canada; Montreal Heart Institute, Montreal, QC H1T 1C8, Canada; Université de Montréal Beaulieu-Saucier Pharmacogenomics Centre, Montreal, QC H1T 1C8, Canada; Montreal Heart Institute, Montreal, QC H1T 1C8, Canada; Department of Medicine, Faculty of Medicine, Université de Montréal, Montreal, QC H3T 1J4, Canada; Montreal Heart Institute, Montreal, QC H1T 1C8, Canada; Université de Montréal Beaulieu-Saucier Pharmacogenomics Centre, Montreal, QC H1T 1C8, Canada; Department of Medicine, Faculty of Medicine, Université de Montréal, Montreal, QC H3T 1J4, Canada

## Abstract

Establishing the relationship between protein-coding genes and phenotypes has the potential to inform on the molecular etiology of diseases. Here, we describe ExPheWas (exphewas.ca), a gene-based phenome-wide association study browser and platform that enables the conduct of gene-based Mendelian randomization. The ExPheWas data repository includes sex-stratified and sex-combined gene-based association results from 26 616 genes with 1746 phenotypes measured in up to 413 133 individuals from the UK Biobank. Interactive visualizations are provided through a browser to facilitate data exploration supported by false discovery rate control, and it includes tools for enrichment analysis. The interactive Mendelian randomization module in ExPheWas allows the estimation of causal effects of a genetically predicted exposure on an outcome by using genetic variation in a single gene as the instrumental variable.

## Graphical Abstract

**Graphical Abstract:**
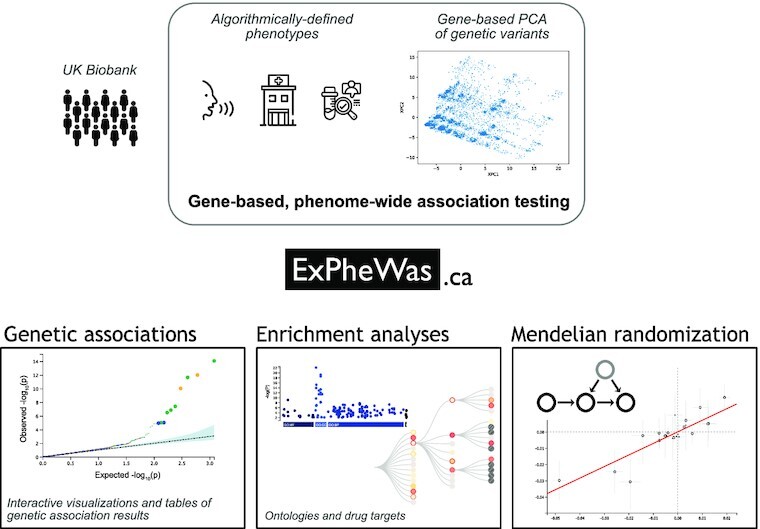
Overview of the ExPheWas study and browser. ExPheWas (exphewas.ca) provides access to gene–phenotype association statistics and enrichment analyses and allows *cis*-Mendelian randomization estimation.

## INTRODUCTION

Uncovering the relationship between genes and phenotypes is an important goal of genetics to help improve our understanding of molecular physiology and disease pathology. Genetic variation provides a tool for predicting the causal effects of altering the functions of a protein and is a valuable approach to help in the validation of drug targets. Phenome-wide association studies (PheWAS) are widely used to study the association of genetic variants across multiple traits and diseases (1,2). Many large databases of single common genetic variant association results have now been made available, thanks to the democratization of genomic datasets and the emergence of high-quality software and web services. For example, PheWeb provides a user-friendly web interface to query GWAS (genome-wide association studies) results, and many independent instances of the software can now be found online (3). Other portals for common genetic variants include PhenoScanner, the GWAS Catalog and the Open Targets Genetics Platform (4–8). Recently, results from rare genetic variants captured by exome sequencing in a phenome-wide approach were also made available in portals such as genebass and the AstraZeneca PheWAS Portal (9).

To date, most PheWAS have focused on the investigation of individual common single-nucleotide polymorphisms (minor allele frequency >1%) or gene-level results based on aggregated rare variants. Here, we fill the information gap with an approach that provides gene-level information based on common genetic variants. We used an association test that relies on principal component analysis (PCA) to aggregate common variants within gene regions (10,11). In cases where multiple genetic variants with individually small effect sizes underlie an association signal, such tests are expected to have greater power than their single-variant counterparts.

Genetic variation can be used to predict the causal effect of intervening on a target exposure under the framework of Mendelian randomization (MR) (12). For example, one may be interested in predicting the effect of a one unit reduction in LDL (low-density lipoprotein) cholesterol on myocardial infarction. This can be achieved, under causal and statistical assumptions, by relating the effect of genetic variation on the exposure (i.e. LDL cholesterol) to their effect on the outcome (i.e. myocardial infarction). Some online tools and browsers have been developed to facilitate MR studies, such as MR-Base that supports the use of different estimators and provides access to a diverse set of summary statistics that can be used to derive the MR estimates interactively (13).

**Figure 1.**
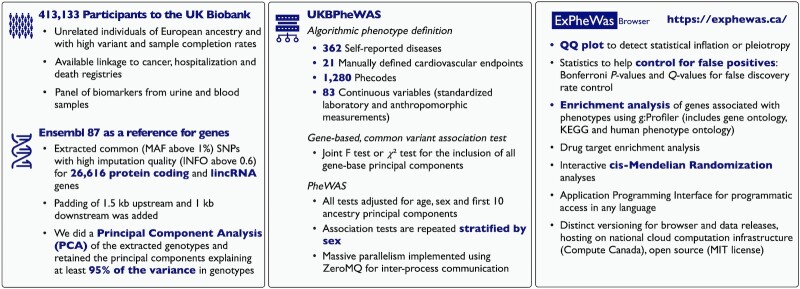
Summary of the ExPheWas analysis and browser functionality.

In this article, we present ExPheWas, a data repository of gene–phenotype association results and a web-based tool for data exploration and gene-based MR analyses (Figure 1). Importantly, ExPheWas also presents both sex-combined and sex-stratified association results to enable the conduct of research that is sensitive to differences between the sexes. ExPheWas can be accessed using the web interface or programmatically, through an application programming interface (API).

## MATERIALS AND METHODS

### UK Biobank and genetic quality control

The UK Biobank is a longitudinal population cohort of >500 000 individuals. All participants visited a recruitment center between 2006 and 2010 and completed a touchscreen-based questionnaire followed by a verbal interview with a nurse allowing participants to self-report a wide variety of diseases. Urine and blood samples were collected allowing for the measurement of an extensive panel of biochemical markers. Linkage to national cancer, hospitalization and death records enables the algorithmic definition of various health-related outcomes. Genetic data derived from a genome-wide genotyping array were also collected and imputed to ∼96 million genetic variants (14).

Because of the high-throughput nature of our study, we conducted a strict genetic quality control to reduce the risk of bias due to poor genotyping, low imputation quality or population stratification. We excluded all variants and individuals with >2% of data values missing. To avoid bias due to cryptic relatedness, we randomly selected one individual from pairs predicted to be related using a kinship coefficient corresponding to a third-degree relationship (0.0884) as a threshold, and we included only individuals from the largest genetically homogeneous population in the UK Biobank corresponding to individuals of European ancestry. We excluded individuals from non-European ancestry based on self-reported data and based on outliers from a manually defined cluster in the genome-wide PCA plot. After these steps, a total of 413 133 individuals remained for analysis.

### Creation of gene-based PCs

We used the Ensembl 87 database as a reference for human protein and lincRNA (long intergenic noncoding RNA) genes. The rationale for including lincRNA genes was to increase genomic coverage and to optimize the inclusion of functional genes. In total, 26 616 genes were analyzed. To create a compact representation capturing genetic variability within gene regions, we conducted PCA with genetic variants of minor allele frequency ≥1% and an imputation quality info score ≥0.6 at every locus. We used the Ensembl gene boundaries and added a padding of 2.5 kb (1.5 kb upstream and 1 kb downstream) to coding genes, but not for lincRNA genes. We extracted all additively encoded genotype dosages from the UK Biobank v3 imputed genotype data release and conducted a PCA using the implementation from scikit-learn in the Python programming language (15). The projection on the space spanned by the PCs retaining 95% of the variance was saved for all samples. In a sensitivity analysis, we also considered retaining 99% of the variance for a subset of genes and this choice had a limited effect on the association test results, as seen in an earlier version of ExPheWas (v0).

### PheWAS

#### Phenotype definition

We included 362 self-reported diseases recorded as part of the verbal interview with a nurse at the baseline visit (variable #20002). We included 83 standardized continuous variables corresponding to anthropometric measurements, and blood and urine biomarkers. Details on transformations used to normalize the continuous variables are provided in Supplementary Table S1. ICD10 codes from the hospitalization and death records were converted to ‘Phecodes’, phenotypic categories that are clinically meaningful and suitable for high-throughput association testing, resulting in 1280 phenotypes (2). Finally, we manually defined 21 cardiovascular endpoints, including myocardial infarction, angina, cardiovascular death and revascularization procedures (see definitions in Supplementary Table S2).

#### Association testing

Different approaches have been proposed to conduct association testing of common variants at a gene locus (11,16,17). Because our PheWAS involves a large number of tests, we used a dimensionality reduction approach to reduce the computational burden and opted for a PCA-based association model that was previously described and that is also used in the MAGMA software (10,11).

For continuous traits, an *F*-test is used to compare two nested models as in MAGMA. First, a null model regresses the outcome on covariates such as age, sex and genome-wide PCs to adjust for confounding due to ancestry. The association test is based on the gain in goodness of fit (or lack thereof) after adding gene-based PCs to the null model. More formally, given two nested models with *P*_1_ and *P*_2_ representing the set of parameters where *P*_1_ ⊂ *P*_2_, one can compute the *F*-statistic given by

(1) $$\begin{equation*} F = \left( \frac{\text{RSS}_1 - \text{RSS}_2}{|P_2| - |P_1|} \right) \div \left( \frac{\text{RSS}_2}{n - |P_2|} \right), \end{equation*}$$

where RSS_*i*_ and |*P*_*i*_| are the residual sum of squares and number of parameters of the *i*th model, respectively, and *n* is the number of samples. This statistic follows an *F*-distribution with (|*P*_2_| − |*P*_1_|, *n* − |*P*_2_|) degrees of freedom under the null hypothesis that the second model does not improve the residual sum of squares.

A similar approach for generalized linear models has been described based on the difference in deviance and was termed the analysis of deviance (18). The difference between the deviances of the nested models follows a *χ*^2^ distribution with |*P*_2_| − |*P*_1_| degrees of freedom under the null hypothesis. This extension can be used in the context of logistic regression as implemented in our analyses. In this setting, an equivalent approach based on the likelihood ratio test was also previously suggested (10).

The *F*-test and analysis of deviance-based methods are available in R under the ‘anova’ function that takes fitted models as parameters (i.e. output from the *lm* or *glm* functions). Because of the large number of tests in our analysis, we used the optimized fastglm (v0.0.1) package when fitting logistic regression models for binary outcomes (github.com/jaredhuling/fastglm). This package uses the Cholesky decomposition to optimize the iterative reweighted least squares algorithm that drastically reduced the time required for logistic regressions. The phenotype coding and statistical tests are implemented in UKBPheWAS, a tool we developed specifically for this analysis (github.com/legaultmarc/ukbphewas).

#### False positive control

In the PheWAS study, we conducted ∼140 million association tests. We used a false discovery rate (FDR) approach to control for false positive results attributable to multiple hypothesis testing, on a per gene and per phenotype basis. Specifically, we used the test *P*-values to compute corresponding *q*-values designed so that if all *q*-values ≤0.05 are considered significant, then 5% of the significant tests will be false discoveries on average (19). For example, when browsing results for a gene of interest, the displayed *q*-values will be based on 1746 tested phenotypes. Browsing the same results by ‘outcome’ will result in *q*-values based on all tested genes. The uncorrected *P*-value and the Bonferroni corrected *P*-value are also provided with each result. When browsing association results for a selected gene, a quantile–quantile (QQ) plot of association *P*-values is also displayed on the results browser along with the *λ* inflation factor corresponding to the ratio of the median observed association statistic to the median expected association statistic under the null. The inflation factor is a quantitative estimate of the deviation of the statistic distribution from the null and high *λ* (>1) values are characteristic of genes that contribute to many phenotypes (i.e. pleiotropic loci). For example, the *HLA-DQB1* gene is notoriously pleiotropic and has *λ* = 2.48.

### Browser and API

#### Implementation

The PheWAS results are accessible via an API and through a web-based results browser, both developed using the Python programming language (v3.8) and Flask web framework (v1.1.1). We used the SQLAlchemy (v1.3.13) object relational mapper and SQL engine to build the results database and the publicly available instance uses PostgreSQL (v10.19). The code for the database models, API endpoints, web browser endpoints and the JavaScript frontend are publicly available online (github.com/pgxcentre/ExPheWAS). Interactive visualizations integrated in the web application were developed using D3.js (d3js.org).

To ensure long-term availability, the service is hosted on the Compute Canada Cloud service (computecanada.ca/home).

#### Enrichment analyses

We integrated enrichment analysis utilities to the API and results browser. For any selected phenotype, it is possible to test whether the associated genes are enriched in drug targets. To achieve this, we used the ChEMBL database to map drug target genes to Anatomical Therapeutic Chemical (ATC) classification codes representing drug classes (20). The ATC codes are structured hierarchically in a five-level system where the first level indicates the anatomical group (e.g. C represents drugs acting on the ‘cardiovascular system’) and the fifth level represents individual drugs (e.g. C07AB07 represents bisoprolol, a specific beta-blocker molecule). For enrichment analyses, we used Fisher’s exact test of the 2 × 2 contingency table of the number of genes associated with the phenotype and drug class at a *q*-value ≤0.05 level.

The drug target enrichment analysis results are displayed on the ExPheWas browser results page as an interactive tree with collapsible nodes. A color scale is used to represent enrichment *P*-value. For every ATC node, the fill color represents the node’s enrichment *P*-value and the stroke color represents the minimum *P*-value in the subtree rooted at the current node.

Enrichment analyses with the g:Profiler API are conducted automatically if over five genes are significantly associated with a phenotype (at FDR 5%) (21). The enrichment results are displayed for KEGG pathways, Gene Ontology and Human Phenotype Ontology terms.

### Mendelian randomization

MR is used to estimate the causal effect of an exposure (*X*) on an outcome (*Y*) using genetic variation as an *instrumental variable*. The popular inverse variance weighted (IVW) estimator combines the causal estimates from different genetic instruments by their precision ($1/{\rm se}(\hat{\beta }_{Yk})^2$), in a manner inspired by fixed-effect meta-analysis methodology (22). Concretely, the IVW estimator for *k* genetic instruments is

(2) $$\begin{equation*} \hat{\beta }_{\text{IVW}} = \frac{\sum _k \hat{\beta }_{Xk} \hat{\beta }_{Yk} {\rm se}(\hat{\beta }_{Yk})^{-2}}{\sum _k \hat{\beta }_{Xk}^2 {\rm se}(\hat{\beta }_{Yk})^{-2}}, \end{equation*}$$

where $\hat{\beta }_{Xk}$ and $\hat{\beta }_{Yk}$ are estimated coefficients for the effect of the *k*th instrument on the exposure and outcome, respectively. In ExPheWas, we use a gene’s PCs as the *k* instrumental variables in this estimator. By default, PCs not associated with the exposure (*P* > 0.05) are removed from the IVW analysis to satisfy the *relevance* assumption (see Supplementary Data). To further help users validate this assumption, we also display the association *P*-value between the selected gene and the exposure. We provide a discussion of the instrumental variable assumptions and specific recommendations in the Supplementary Data

Because the MR estimates are based only on a single gene of interest, they can be interpreted within the *cis*-MR framework (23) (see Supplementary Data). Because we do not have access to direct measurements of the gene products in this study, users should select exposures that are biologically or mechanistically related to their gene of interest. For example, if one was to conduct a *cis*-MR based on the *PCSK9* gene, a protein involved in the recycling of the LDL receptor, levels of LDL cholesterol would be a good choice of proxy phenotype for *PCSK9* function.

## RESULTS AND DISCUSSION

### PheWAS

To test the association between genes and multiple phenotypes, we used a PheWAS approach (1,2). We tested the association between 26 616 protein-coding and lincRNA gene regions and 1746 phenotypes in the UK Biobank. We included phenotypes spanning a large proportion of the phenome and defined from different data sources available in the UK Biobank. We used Phecodes based on the hospitalization and death records to capture many clinically meaningful phenotypes, as well as continuous measurements from the blood or urine assays, anthropometric measurements, self-reported traits or manually defined cardiovascular outcomes.

All analyses were also conducted in male-only and female-only subgroups that were genetically determined according to X and Y chromosomes. The availability of sex-stratified results allows for the detection of associations between genes and phenotypes that have sex-specific effects and supports the conduct of sex-specific causal analyses using the MR module.

### Browser

The web-based browser (exphewas.ca) was developed to enhance the dissemination of results from the gene-based PheWAS analysis and to facilitate interpretation of the results. The interface was designed to be user friendly while supporting programmatic access, as the website itself uses the publicly available API to access all of its data. The browser is thoroughly documented and offers three main modules.

**Figure 2.**
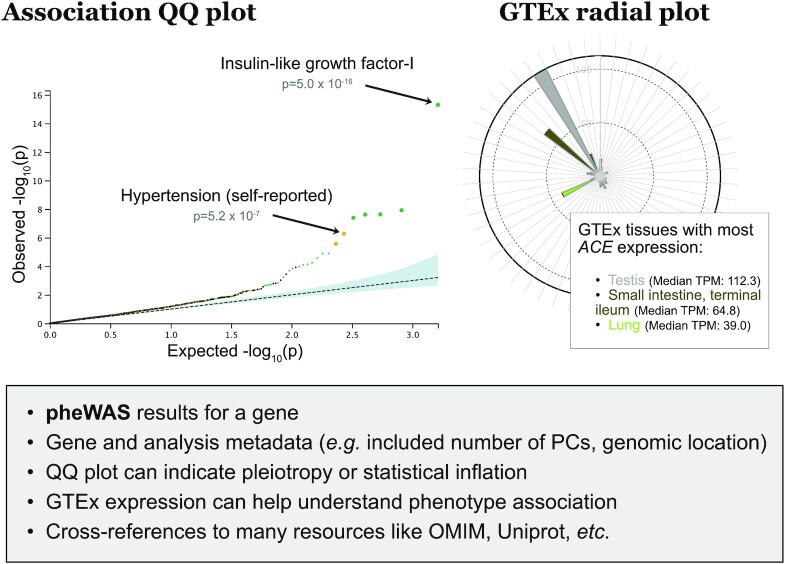
Interactive visualizations on the ‘gene’ pages. The example is for the angiotensin I converting enzyme (ACE). (Left) A QQ plot representing deviation from the expected distribution of *P*-values under the null hypothesis. The statistical inflation as measured by *λ* is 1.22 in this example (shown on the website). (Right) A radial plot of median expression in GTEx v8 tissues, which can be used to contextualize association results.

First, the gene page (summarized in Figure 2) allows users to search for and select a gene of interest and to access the PheWAS page reporting association results with all phenotypes. Interactive visualizations on the gene pages include the gene expression data from GTEx showing median expression across tissues that can inform users of the biological plausibility of the association results as well as a QQ plot that can help diagnose statistical inflation due to latent confounding or polygenicity.

**Figure 3.**
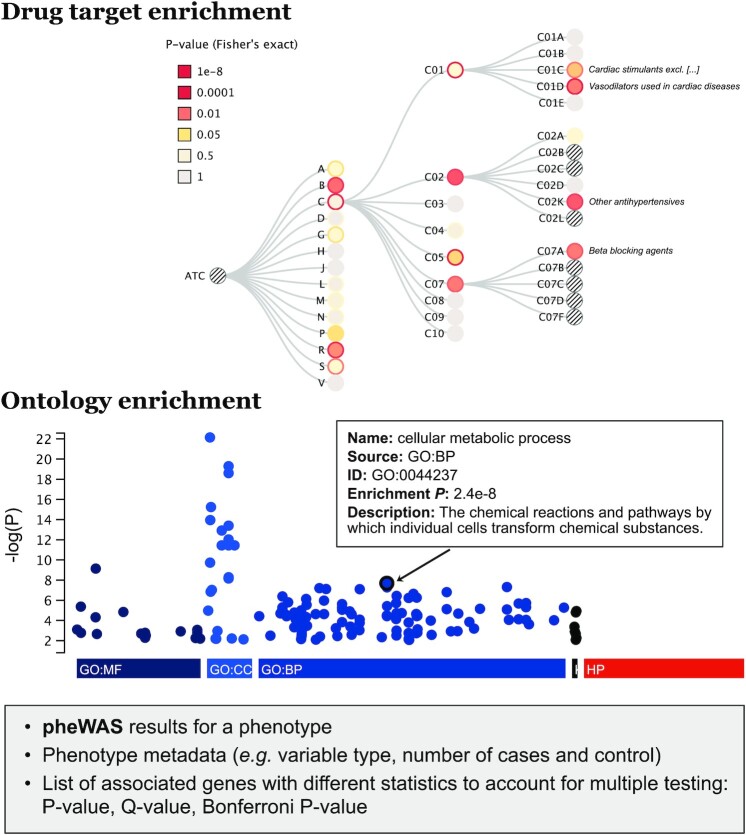
Overview of the enrichment analyses presented on ‘phenotype’ pages. The example is for the ‘essential hypertension’ (401.1) phenotype. (Top) Results from a drug target enrichment analysis using Fisher’s exact test. (Bottom) Enrichment analyses for the Gene Ontology molecular functions (GO:MF), cellular components (GO:CC) and biological processes (GO:BP), the KEGG pathways and the Human Phenotype Ontology. Enrichment results are fetched dynamically from g:Profiler when enough significantly associated genes are available for analysis (at FDR 5%).

Second, the phenotype page (summarized in Figure 3) allows users to search for phenotypes and access a page showing all gene association results. The phenotypes are categorized into four data types: Phecodes, algorithmically defined cardiovascular endpoints, self-reported diseases and continuous variables. This page also includes enrichment analyses with drug targets of different drug classes defined according to the ATC classification. For example, in ExPheWas, the genes associated with self-reported heart attacks (phenotype #1092) are enriched for genes that encode drug targets for ‘lipid modifying agents’ (ATC code #C10A) and the Fisher’s exact test *P*-value is 0.011. When five or more genes are associated with a selected phenotype at an FDR threshold of 0.05, the g:Profiler is automatically used to test for enrichment within the KEGG pathways, Gene Ontology and the Human Phenotype Ontology terms (21).

**Figure 4.**
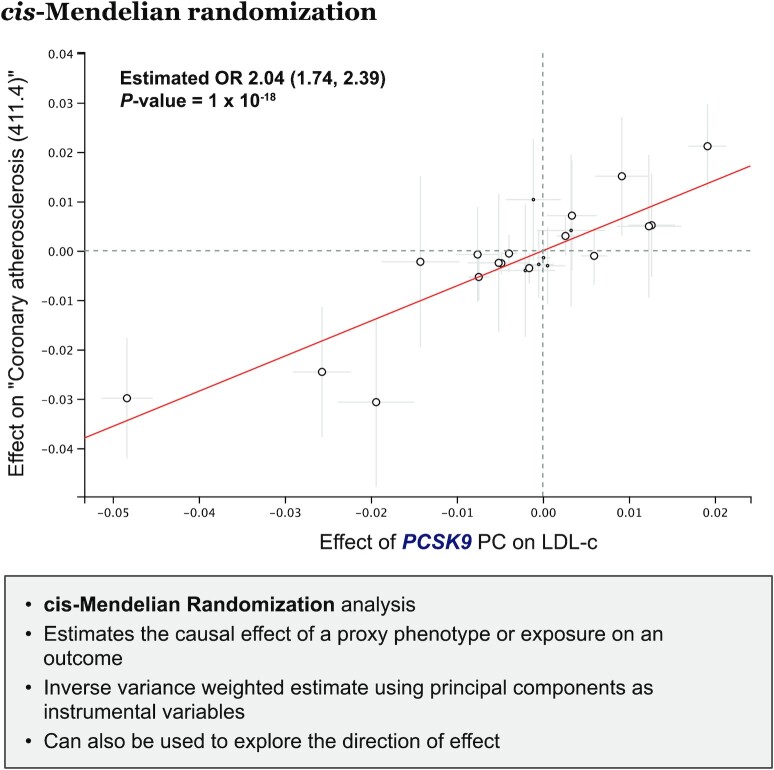
Overview of the *cis*-MR analysis module. The example shows the estimated causal relationship for a one standard deviation reduction in LDL cholesterol through a genetic disruption of the *PCSK9* gene on the odds of ‘coronary atherosclerosis’ (411.4). Smaller points (near the dashed vertical line) represent PCs with a null effect (*P* > 0.05) on the exposure that were excluded from the IVW estimate in order to satisfy the *relevance* assumption. The slope of the red line corresponds to the IVW estimate.

Finally, to allow users to conduct a deeper interpretation of the PheWAS results, we implemented a *cis*-MR module (Figure 4). This module allows users to estimate the causal effect (under the instrumental variable assumptions) of an exposure on a phenotype where the exposure is a selected phenotype modulated by genetic variation at a gene of interest. This module provides an answer to research questions such as ‘What would be the effect of a one standard deviation reduction in LDL cholesterol levels derived from genetic variants encoding the protein targeted by statins (the *HMGCR* gene) on myocardial infarction?’.

A versioning system is used to ensure that results accessed through the browser remain stable. The interface/software is versioned separately from the data analysis version. The current data release dates from 13 October 2021 and is labeled ‘v1’. Even though the home page corresponds to the latest data release, the previous data release remains available under a stable URL: exphewas.ca/v0. Browser versions are indicated in the top right corner of the web page and are composed of a major, a minor and a micro version digit. We reserve major digit version changes for API breaking changes (e.g. a change in data release), minor digit changes for significant new features or critical bug fixes, and the micro digit for smaller changes. The data and browser are hosted on the Compute Canada Cloud service platform.

### Example applications

#### Investigation of atrial fibrillation genes

Atrial fibrillation is a common cardiac arrhythmia of the atria that can lead to severe complications, including stroke, cardiomyopathy and heart failure (24,25). We used PheWAS analysis results (data release v1.0) that included 18 474 UK Biobank participants with atrial fibrillation established based on hospitalization and death records (Phecode #427.2, ‘atrial fibrillation and flutter’). There were 400 genes associated with atrial fibrillation with a *q*-value ≤0.05. The automated g:Profiler-based enrichment tool found enrichment for supraventricular arrhythmia (Human Phenotype Ontology, HP:0005115; *P*_adj_ = 1.2 × 10^−6^), cardiac muscle contraction (Gene Ontology biological process, GO:0060048; *P*_adj_ = 3.9 × 10^−6^) and Z disk (Gene Ontology cellular component, GO:0030018; *P*_adj_ = 1.7 × 10^−4^), among other relevant terms.

The enrichment of atrial fibrillation-associated genes in drug targets based on the ChEMBL database (20), as implemented in ExPheWas, found enrichment for class Ia and class Ib antiarrhythmics corresponding to ATC codes C01BA and C01BB, respectively. The Fisher’s exact test enrichment *P*-values for these classes were 0.032 for both. This finding is concordant with pharmacological treatment for atrial fibrillation suggesting genetic support for these drug targets.

We further explored gene associations that were not previously reported in the GWAS Catalog. Notably, myotilin encoded by the *MYOT* gene was associated with heart rate (*P* = 2.9 × 10^−32^) and atrial fibrillation (*P* = 2.2 × 10^−16^) in our PheWAS. This region is located in a long LD block spanning multiple genes (Supplementary Figure S1). Other credible genes that could drive the association signal in this region are *FAM13B* (atrial fibrillation, *P* = 1.3 × 10^−14^), *PKD2L2* (*P* = 1.4 × 10^−14^), *WNT8A* (*P* = 8.8 × 10^−17^) and *NME5* (*P* = 4.6 × 10^−9^). The two top genes according to our analysis are *MYOT* and *WNT8A*, which have *P*-values two to three orders of magnitude smaller than the others. However, *WNT8A* is not expressed in heart tissues in GTEx, whereas *MYOT* is expressed in the heart. Myotilin is a component of the sarcomeric Z disk, a structure implicated in muscle contraction, and rare mutations in myotilin cause myofibrillar myopathy, which often co-occurs with cardiomyopathy [OMIM:604103 (26,27)]. In single-cell RNA sequencing analysis, *MYOT* was found to be expressed in left atrial and right ventricular cardiomyocytes in line with atrial fibrillation pathophysiology (28). Even though it was located nearby, we did not consider *KLHL3* as leading the association signal observed with *MYOT*, as it showed an independent association signal through stepwise conditional analysis (Supplementary Figure S1). After two stages of the forward conditional stepwise analysis, no further variants remained associated (Supplementary Figure S1).

## CONCLUSION

We have presented ExPheWas, a data repository of gene–phenotype association results and a web-based tool for data exploration and gene-based MR analyses. ExPheWas can be accessed using the web interface or programmatically, through an API. Both sex-combined and sex-stratified association results are provided to support the conduct of research that is sensitive to differences between the sexes.

ExPheWas can be used in multiple applications, including for follow-up of GWAS findings and for drug target validation. The gene-based approach can provide more statistical power than single-variant analysis, and facilitates biological interpretation through easier integration with gene-level annotations such as ontological terms. Importantly, the provided MR platform supports the investigation of the causal relationship between selected phenotypes.

The analytical approach behind ExPheWas does have some limitations worth mentioning. First, overlapping genes within the boundaries of a protein-coding gene and linkage disequilibrium may result in spurious attribution of gene–phenotype associations. Information on the functional characteristics of genetic variants would be needed to help untangle such associations. Additionally, to avoid bias due to population stratification and to maximize statistical power, we conducted our analysis in the largest homogeneous subgroup of individuals in the UK Biobank comprising participants of European ancestry. This selection may hamper the transferability of association results to populations with different ancestries.

To conclude, the ExPheWas browser and platform contributes an important atlas of gene–phenotype associations along with tools to interrogate, contextualize and interpret the results. ExPheWas enables gene-level discoveries that may otherwise have been missed by traditional single-variant approaches. Furthermore, the integrated *cis*-MR module provides a powerful tool to conduct causal inference and to further our understanding of gene-specific causal effects.

## DATA AVAILABILITY

Access to the UK Biobank resource requires application through the access management system and instructions are available online: https://www.ukbiobank.ac.uk/enable-your-research/apply-for-access. The ExPheWas results browser is available online at https://exphewas.ca/. The code for the results browser including the database models, the web application and the data visualizations is open source and available at https://github.com/pgxcentre/ExPheWAS.

## Supplementary Material

gkac289_Supplemental_Files
