# Supplementary material for: ExPheWas: a platform for cis-Mendelian randomization and gene-based association scans
Source: Nucleic Acids Res. 2022 Apr 26;50(W1):W305–11. doi: 10.1093/nar/gkac289 (PMC9252780; doi:10.1093/nar/gkac289)
Supplement: gkac289_Supplemental_Files [file gkac289_supplemental_files.zip › exphewas_supplementary_material.pdf]

Supplementary Material

ExPheWas Mendelian randomization analyses

Assumptions

In ExPheWas, we implemented a Mendelian randomization (MR) estimator that relies on instrumental variable assumptions. These assumptions are commonplace in the MR literature and are mentioned below:

1. **Relevance**: The instrumental variable is associated with the exposure.
2. **Unconfoundedness**: There are no common causes of the genetic instrumental variable and outcome.
3. **Exclusion**: The effect of the instrumental variable on the outcome is fully mediated by the exposure.

In ExPheWas, the gene-based principal components are used as instrumental variables.

The first assumption (*relevance*, 1.) can easily be verified from the data and the gene-exposure p-value is automatically presented in ExPheWas to assess this criterion. Furthermore, individual principal components with P-values above 0.05 for association with the exposure are automatically excluded as genetic instruments, and users also have the option to retain all principal components in the analysis.

The second and third assumptions are unverifiable from the data alone. Violations of the *unconfoundedness* assumption (2.) are typically due to population stratification involving the influence of differential allele allocations. Population stratification is controlled for in ExPheWas through the use of an ancestrally homogeneous subset of participants to the UK Biobank and with the adjustment in all regression models for the genome-wide principal components. The magnitude of bias due to residual fine scale population structure is expected to be minimal.

The third criterion (*exclusion*, 3.) is typically the most problematic. It is possible that a causal path independent of the exposure exists if the genetic region used for the analysis is in linkage disequilibrium (LD) or includes functional elements unrelated to the gene of interest. The

gene of interest could also be pleiotropic (*i.e.* it could involve biological pathways linked to the outcome other than the one acting through the selected exposure). Pleiotropy can be assessed in ExPheWas by investigating the causal effects on an outcome acting through exposures based on other phenotypes that are associated with the gene of interest. However, the effect of LD or co-localizing elements is more pernicious and requires investigators to support their observations using complementary approaches and to be careful in their interpretation of results (see the Specific recommendations section).

cis-MR and drug target MR

When MR is used to predict the results of clinical trials, it is advised to consider the effect of genetic variants in the gene encoding the drug target of interest as other genetic mechanisms that modulate the same outcome may have subtly different clinical effects. More concretely, to predict the effect of PCSK9 inhibitors, a drug class lowering low density lipoprotein cholesterol (LDL-c), it is reasonable to only include genetic variants at the *PCSK9* gene locus in a MR study. This concept, where only variants acting in *cis* of the target gene are of interest, has been more formally described in Schmidt *et al.* [1] The authors emphasize that although it would be ideal to have direct measurements of the gene product enabling formal mediation analysis when considering the effect of a specific gene, it is also possible to use proxy phenotypes selected based on the known gene function in order to estimate the magnitude of the effect of genetic perturbation on the target gene. The implementation of MR in ExPheWas was designed under the proxy paradigm.

Specific recommendations

1. **Ensure that the selected gene–exposure pair has a robust and well understood relationship.** For example, converging evidence from different sources (*e.g.* GWAS, family studies, Mendelian diseases, model organisms or studies of predicted loss-of-function variants) adds credibility to a result. The gene and exposure should also be strongly associated in ExPheWas.
2. **Assess the possibility of confounding due to LD or overlapping genetic features**. It may help to visualize overlaping genes in the reported region, and to assess results derived from gene regions centered on neighbouring genes.
3. **Assess the risk of violations of the exclusion assumption through pleiotropic mechanisms**. Visualizing the gene association QQ-plot (available on the gene pages in ExPheWas) is a good way to rapidly evaluate the risk for pleiotropy. Note that many associated phenotypes within the *same causal path*, so called "vertical pleitropy" is not problematic in MR.

Supplementary Methods References

1. Schmidt, A. F. *et al.* Genetic drug target validation using Mendelian randomisation. *Nat Commun* **11,** 3255 (June 2020).

Supplementary Figures

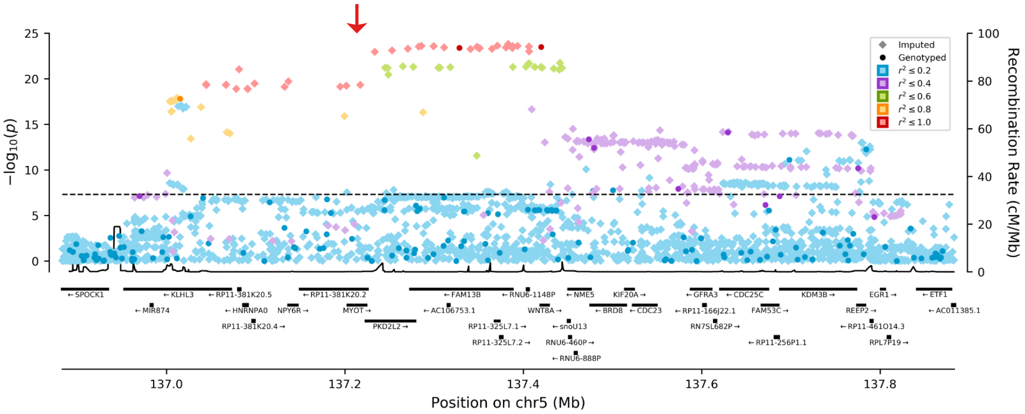

(a) Initial scan conditional only on age, sex and the first 10 PCs.

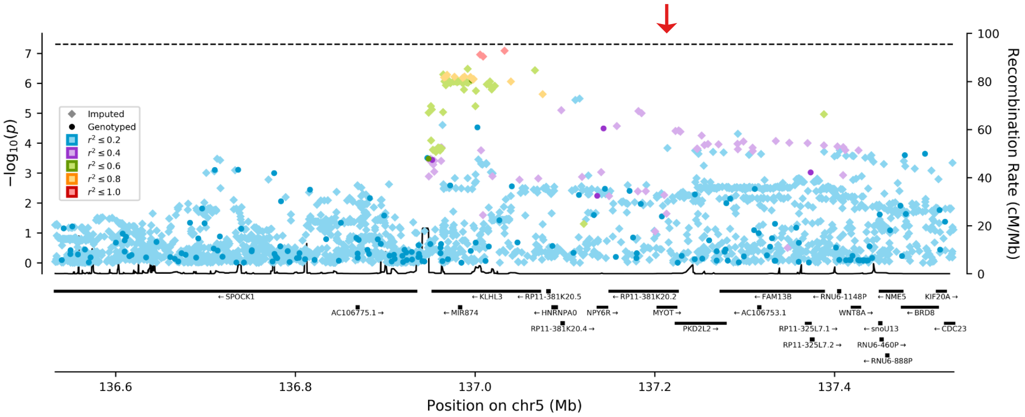

(b) Association scan conditional on the lead variant from stage 1, rs148378888.

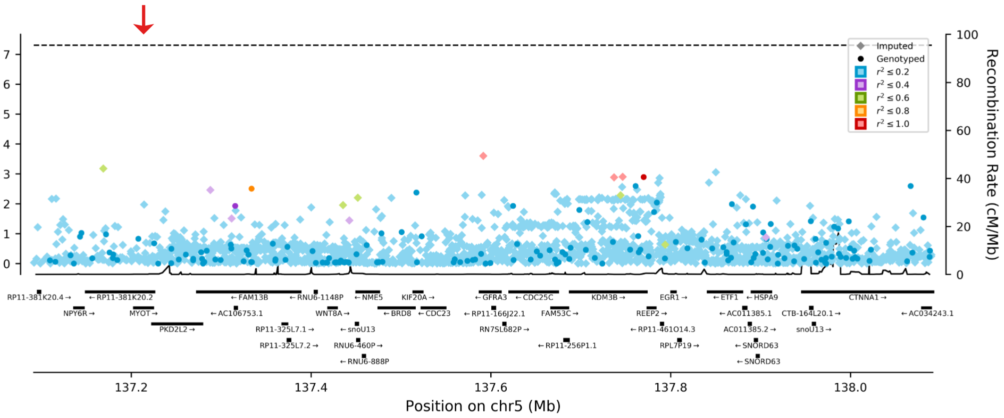

(c) Association scan conditional on the lead variants from previous stages: rs148378888 and rs12653760.

Supplementary Figure 1: **Stepwise forward conditional analysis of atrial fibrillation associated variant at the chr5:136,883,078-137,883,078 (GRCh37) locus in the UK Biobank.** The red arrows indicate the position of the *MYOT* gene.

Supplementary Tables

Supplementary Table 1: Summary of the included continuous variables and transformations used to obtain approximately normally distributed variables.

[This table is provided as an Excel file]

Supplementary Table 2: **Definition of the algorithmically-defined outcomes.**

**Outcome**	**Definition***
Death outcomes	
Any death	Any entry in the death records variable #40001
Cardiovascular death	Any "I" code as the primary cause of death
Coronary artery disease death	I20–I25 as the primary cause of death
Cardiovascular composite outcomes	
Myocardial infarction	ICD9 codes: 410, 412, 411.0, 429.79<br>ICD10 codes: I21, I22, I23, I25.2 in the hospitalization or death records
Percutaneous coronary intervention / Coronary artery bypass graft (PCI/CABG)	OPCS procedure codes: K40, K41, K42, K43, K44, K45, K46, K49, K50, K75
Unstable angina	I20.0 code as the primary reason for hospitalization or cause of death
Any angina	ICD9 code 413 or ICD10 code I20 in the hospitalization or death records
Coronary artery disease	ICD9 codes: 410–414 except for aneurysms (414.1)<br>ICD10 codes: I20–I25 in the hospitalization or death records or operation codes for PCI/CABG as previously fined
Heart failure	ICD9 codes: 425, 428<br>ICD10 codes: I42, I50 in the hospitalization or death records

* Unless otherwise specified, codes were taken in both the primary and secondary reasons for hospitalization, but only the primary cause of death was used.
